# Molecular Phylogeny of *Gueldenstaedtia* and *Tibetia* (Fabaceae) and Their Biogeographic Differentiation within Eastern Asia

**DOI:** 10.1371/journal.pone.0162982

**Published:** 2016-09-15

**Authors:** Yan-Ping Xie, Ying Meng, Hang Sun, Ze-Long Nie

**Affiliations:** 1 Key Laboratory for Biodiversity and Biogeography of East Asia, Kunming Institute of Botany, Chinese Academy of Sciences, Kunming, Yunnan, China; 2 University of Chinese Academy of Sciences, Beijing, China; 3 Key Laboratory of Plant Resources Conservation and Utilization, College of Biology and Environmental Sciences, Jishou University, Jishou, Hunan, China; Institute of Botany, CHINA

## Abstract

*Tibetia* and *Gueldenstaedtia* are two morphologically similar and small genera in Fabaceae, with distributions largely corresponding to the Sino-Himalayan and Sino-Japanese subkingdoms in eastern Asia, respectively. These two genera have confusing relationships based on morphology; therefore, we aimed to provide a clear understanding of their phylogenetic and biogeographic evolution within eastern Asia. In our investigations we included 88 samples representing five *Gueldenstaedtia* species, five *Tibetia* species, and outgroup species were sequenced using five markers (nuclear: ITS; chloroplast: *matK*, *trnL-F*, *psbA-trnH* and *rbcL*). Our phylogenetic results support (1) the monophyly of *Tibetia* and of *Gueldenstaedtia*, respectively; and (2) that *Tibetia* and *Gueldenstaedtia* are sister genera. Additionally, our data identified that *Tibetia* species had much higher sequence variation than *Gueldenstaedtia* species. Our results suggest that the two genera were separated from each other about 17.23 million years ago, which is congruent with the Himalayan orogeny and the uplift of the Tibetan Plateau in the mid Miocene. The divergence of *Tibetia* and *Gueldenstaedtia* is strongly supported by the separation of the Sino-Himalayan and Sino-Japanese region within eastern Asia. In addition, the habitat heterogeneity may accelerate the molecular divergence of *Tibetia* in the Sino-Himalayan region.

## Introduction

*Gueldenstaedtia* Fischer is a small genus in the legume family (Fabaceae) which was named by F. E. Fischer to pay tribute to the Russian naturalist Gueldenstaedt [1]. This genus has usually been divided into two subgenera based on morphological characteristics of the stipules, style, and seeds: subgenera *Gueldenstaedtia* and *Tibetia* [2]. Species in subgenus *Gueldenstaedtia* are characterized by lateral stipules that are not leaf-opposed, free from each other, and seeds that are glazed, pitted, uniformly colored, and never spotted. Species in subgenus *Tibetia* has stipules that are amplexicaul, leaf-opposed, and united at least in the young condition, and seeds that are unglazed, never pitted, and have irregular blackish spots (Table 1).

**Table 1:** Morphological, cytological and ecological differences among *Tibetia* and *Gueldenstaedtia* s.str. and *Chesneya* (Fabaceae).

Character	*Tibetia*	*Gueldenstaedtia*	*Chesneya*
Stem	almost absent or very short, sometimes prostrate	almost absent, or very short, with many branches	short, lignified
Stipules	amplexicaul, leaf-opposed, united, at least when young	lateral, not leaf-opposed, free from each other	herbaceous, adnate to petiole
Corolla color	yellow or purple	purple	yellow or purple
Pollen grains	3- and 4-colporate	3-colporate	
Pods	cylindric (blunt at apex)	cylindric or linear (acute at apex)	oblong to linear
Seeds	reniform, unglazed, never pitted, with irregular blackish spots	triangular-reniform, glazed, pitted, uniformly colored, never spotted	reniform
Chromosome number	2n = 16 (x = 8)	2n = 14 (x = 7)	2n = 16 (x = 8)
Elevation	3000–5000 m	100–1600m	2900–5300 m
Distribution	Bhutan, China, India, Nepal, Pakistan;	from Russia (Siberia) to the Sino-Himalayan region	central and southwest Asia, Mediterranean region

However, Tsui [3] considered the morphological variation between these two groups distinct and promoted the subgenus *Tibetia* to a generic level as *Tibetia* (Ali) Tsui. With the exclusion of *Tibetia*, *Gueldenstaedtia* s. str. is composed of 10 species according to Flora Republicae Popularis Sinicae [4]. However, Zhu [5] combined six species (i.e., *G*. *gansuensis* Tsui, *G*. *gracilis* Tsui, *G*. *stenophylla* Bunge, *G*. *delavayi* Franch., *G*. *harmsii* Ulbr., *G*. *maritima* Maxim.) into *G*. *verna* (Geogi) Boriss., because the morphological diagnostic characteristics exhibited considerable variation with the change of habits and seasons. For example, *G*. *stenophylla* differs from *G*. *verna* only in leaf size; *G*. *verna* is distinguished from *G*. *delavayi* and *G*. *gracilis* by plant height and leaf shape during fruiting period. Currently, only four species are recognized (Zhu 2004; i.e., *G*. *henryi* Ulbr., *G*. *taihangensis* Tsui, *G*. *verna*, *G*. *monophylla*) and only three are presented in *Flora of China* (i.e., *G*. *henryi* Ulbr., *G*. *taihangensis* Tsui, *G*. *verna*). The species *G*. *monophylla* was not included in *Flora of China* because it has never been collected in China recently [6].

The newly defined genus *Tibetia* is comprised of five species, but species delimitation and classification remain in dispute. Traditionally, two sections were proposed based on the shape of pollen grains and stipules [3]. *Tibetia* section *Glaberae* contains two species with 3-colporate pollen grains and stipules that are round at the apex. *Tibetia* section *Tibetia* has 4-colporate pollen grains and stipules that are acute at the apex. However, new evidence suggested that *T*. *coelestis*, which has 4-colporate pollen grains should be moved into section *Tibetia* and treated as a variation of *T*. *yunnanensis*. Similarly, *T*. *yadongensis*, which has 3-colporate pollen grains should be a member of the section *Glaberae* rather than the section *Tibetia*. Furthermore, *T*. *liangshanensis* P. C. Li was first described as a new species that has a stem height of 15–25 cm, more leaflets, and purple flowers distinguishing from *T*. *forrestii* [7,8].

*Gueldenstaedtia* was traditionally placed in subtribe Astragalinae of tribe Galegeae [9], but cytological and molecular data suggested that *Chesneya* may be the closest relative of *Gueldenstaedtia* [10–12]. A new tribe, *Caraganeae*, was proposed by two Iranian scholars including *Chesneya* and *Gueldenstaedtia* together with three other genera: *Caragana*, *Halimodendron*, and *Calophaca* [13]. This treatment is consistent with their cytological characteristics, as the chromosome counts of *Caragana*, *Chesneya*, and *Tibetia* are 2n = 16, except for 2n = 14 in *Gueldenstaedtia* [11,14,15]. The *Chesneya* group (including *Chesneya*, *Gueldenstaedtia*, and *Tibetia*) was suggested to be delimitated from tribe Caraganeae, and the status of the *Chesneya* group remains unclear [10].

**Fig 1:**
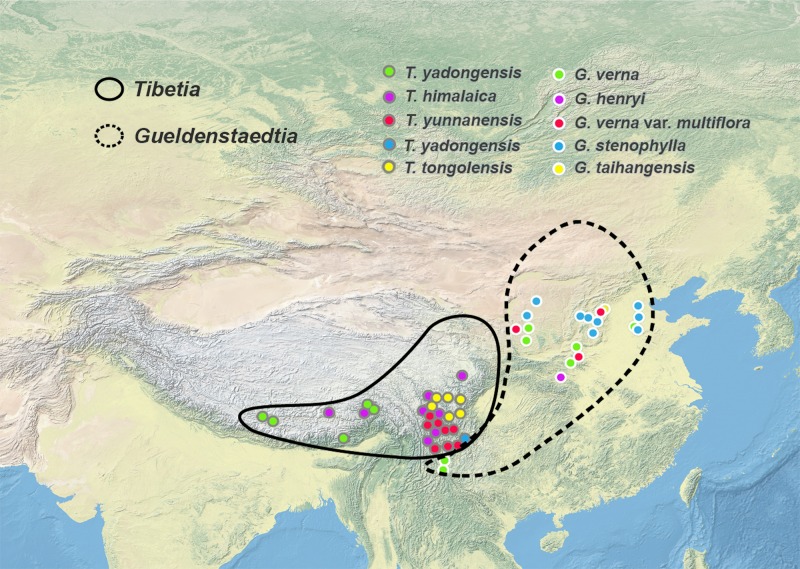
The distribution area and sample sites of *Tibetia* and *Gueldenstaedtia* in eastern Asia. The map is from the website http://www.naturalearthdata.com/.

*Gueldenstaedtia* is restricted to the Sino-Japanese region of eastern Asia. *Gueldenstaedtia* species also occur in the eastern edge of the Qinghai-Tibetan Plateau (QTP), including Gansu, Sichuan, and Yunnan Provinces, but have never been found on the internal QTP and the Himalayan region. In contrast, all of the *Tibetia* species occur in the Himalayan region and the QTP of southwestern China (Fig 1). The distribution boundary between these two genera roughly matched the floristic division between the Sino-Himalayan and Sino-Japanese subkingdoms in the East Asiatic Kingdom [16,17]. Therefore, the *Tibetia*—*Gueldenstaedtia* pair represents a useful example to investigate the correlation between species diversification and floristic heterogeneity on a regional scale.

Previous phylogenetic studies have primarily focused on relationships at the tribal level with only limited sampling of *Tibetia* and *Gueldenstaedtia* [9,10,12]. Many holes remain in our understanding of the infrageneric classification and evolutionary relationships between and within these two genera. A robust estimate of phylogenetic relationships is needed based on extensive sampling that covers their major distribution.

Here we provide the first and most comprehensive phylogenetic analysis of the pair group of *Tibetia* and *Gueldenstaedtia* using data from nuclear ITS, two plastid coding regions, *rbcL* and *matK*, and two non-coding regions, *psbA-trnH* and *trnL-F*, which have been widely used in the legume family [10,18–21]. We intend to: (1) address the monophyly of these two genera, (2) reconstruct phylogenetic species relationships within each genus, and (3) trace the evolution and diversification history of these two genera, especially focusing on their biogeographic correlation to the Sino-Himalayan and Sino-Japanese subkingdoms in eastern Asia.

## Materials and Methods

### Sampling and amplifications

A total of 88 taxa were sampled in the analysis representing all species of the two genera except for *Gueldenstaedtia monophylla* and *Tibetia forrestii* according to the most recent classification system by Zhu [5,22]. *Gueldenstaedtia monophylla* was recorded from Altai mountain area of Russia, Mongolia and China, but we are failed to find it in the field. As for *T*. *forrestii*, it might be a variant of *T*. *yunnanensis* as previously mentioned [8]. Five species of *Chesneya* were used in this study to test its relationship to *Tibetia* and *Gueldenstaedtia*. Outgroup taxa include samples of *Caragana rosea*, *C*. *sinica*, *C*. *microphylla*, *C*. *korshinskii*, *C*. *arborescens*, and *Halimodendron halodendron*, which are from the Caraganeae tribe, and *Hedysarum vicioides* was selected according to the previous molecular phylogeny [21,23]. Collection information is listed in S1 Table. Voucher specimens were deposited in the Kunming Institute of Botany Herbarium (KUN).

Total genomic DNA was isolated from leaf material, using the plant total DNA extraction Kit (BioTeke, Beijing, China) following the manufacturer’s protocol. Five molecular markers (nuclear ITS and plastid DNA sequences from *rbcL*, *matK*, *trnL-F*, and *psbA-trnH*) were sequenced. PCRs were performed in 25-uL reactions using 2 × Power Taq PCR MasterMix (BioTeke, Beijing, China). Typical reaction conditions were as follows: 4 min at 94°C for denaturation, followed by 32 cycles of 40 s at 94°C, 40 s at 55°C for annealing, 1 min 30 s at 72°C for primer extension, then a final 10 min incubation at 72°C. The amplified products were then purified using polyethylene glycol (PEG) precipitation using standard protocols. Cycle sequencing reactions were conducted using BigDye 3.1 reagents on an ABI 3730 automated sequencer (Applied Biosystems, Foster City, California, USA). All sequences are deposited in GenBank (S1 Table).

The original sequences were assembled into contigs and edited using the program Sequencher 4.14. Sequence alignment was performed using ClustalW version 1.8 [24] followed by manual modification in the program BioEdit v7.0.4 [25]. Phylogenetic trees were reconstructed using maximum parsimony (MP), maximum likelihood (ML), and Bayesian inference (BI). Maximum parsimony searches were performed with tree bisection-reconnection branch swapping, with MulTrees on, and simple taxon addition in PAUP* 4.0b10 [26]. Parsimony bootstrap (BP) support [27] for the clades was estimated as described above from 1000 heuristic search replicates, with 100 random taxon addition replicates saving all optimal trees at each step. The ML trees were inferred with RAxML BlackBox, online software based on the so-called rapid bootstrap [28]. Maximum likelihood was implemented starting from random trees, using 1000 rapid bootstrap inferences with RAxML GAMMA model of rate heterogeneity, and ML estimate of alpha-parameter GAMMA Model parameters in effect. Finally draw bootstrap support values on the best-scoring ML tree.

The optimal DNA substitution model was estimated using Akaike information criterion (AIC) in jModeltest 0.1.1 [29,30]. Bayesian inferences were implemented in MrBayes 3.1.2 [31] with models estimated as above. A Bayesian analysis was performed on the condition that the Bayesian Markov Chain Monte Carlo (MCMC) calculation is set to 10,000,000 generations with 4 incrementally heated chains and sampling every 1000 generations. The *Tibetia* species began and convergence estimations were graphically assessed using AWTY [32]. The remaining 9000 trees were sampled from the posterior distribution to calculate the posterior probabilities (PP).

The data were analyzed separately for ITS and for the combined chloroplast dataset. Congruence among the different datasets was first tested using the incongruence length difference (ILD) [33,34] test in PAUP. Taxa with missing data were excluded in the ILD test.

### Divergence time estimation

Due to difficulties in aligning ITS sequences across the diversity of Papilionoideae taxa included in this study and the lack of a sufficient number of other sequences including *rbcL*, *psbA-trnH*, *trnL-F* from taxa representing all groups included in the analyses, *matK* was selected to estimate the origin and diversification time. Previous studies on the legume family have successfully used *matK* sequences for molecular dating [35,36]. Sequences from GenBank representing the main clades in the Papilionoideae were applied in the dating analysis, and *Caesalpinia andamanica* and *Haematoxylum brasiletto* were selected as outgroup taxa. A likelihood ratio test [37] was conducted for clock-like behavior. The results suggested that rate constancy in this data set is not supported at P < 0.05.

BEAST v1.8.0 (http://beast.bio.ed.ac.uk) was used to estimate divergence times [38], which employs a MCMC to co-estimate topology, substitution rates and node ages. All of the analyses were performed based on a General Time Reversible (GTR) nucleotide-substitution model with a Gamma distribution and four rate categories. Divergence times and the corresponding credibility intervals were estimated using a lognormal relaxed molecular clock model and the Birth-Death prior set. Posterior distributions of parameters were approximated using two independent MCMC analyses of 20,000,000 generations with a 10% burn-in. Samples from the two chains that yielded similar results were combined, and convergence of the chains was checked using the program Tracer 1.5 [39]. Samples from posterior distributions were summarized on a maximum clade credibility (MCC) tree with the maximum sum of posterior probabilities on its internal nodes using TreeAnnotator v1.6.1 with the posterior probability limit set to 0.5 to summarize mean node heights. The MCC tree was visualized using FigTree v1.3.1 and the means and 95% higher posterior densities (HPD) were obtained.

The age constraints derived from the legume fossil records were incorporated into the BEAST analysis. The origin time of Papilionoideae was set to 58.6 million years ago (Ma) according to Lavin et al. [35]. There are numerous options from the large fossil record from Papilionoideae, which have been dated to the Late Paleocene and come from America, Europe, Africa, and Asia [40]. Three fossil calibrations were used: (1) *Robinia* stem clade was fixed to 34 (± 0.1) Ma based on a wood fossil possessing apomorphic traits unequivocally related to the *Robinia* stem clade [41–44]; (2) the *Diplotropis* stem clade was set to 56 (± 0.1) Ma, based on a fossil of the leaves and pods that is from the Late Paleocene as well as Middle Eocene, and are very similar to *Bowdichia* [45,46]; (3) the *Styphnolobium* stem clade was set to a constraint of 40.1 (± 0.4) Ma (Middle Eocene), given that *Styphnolobium* and subgenus *Cladrastis* have a fossil of a pod and leaflets from Middle Eocene from Tennessee, USA [40].

## Results

**Fig 2:**
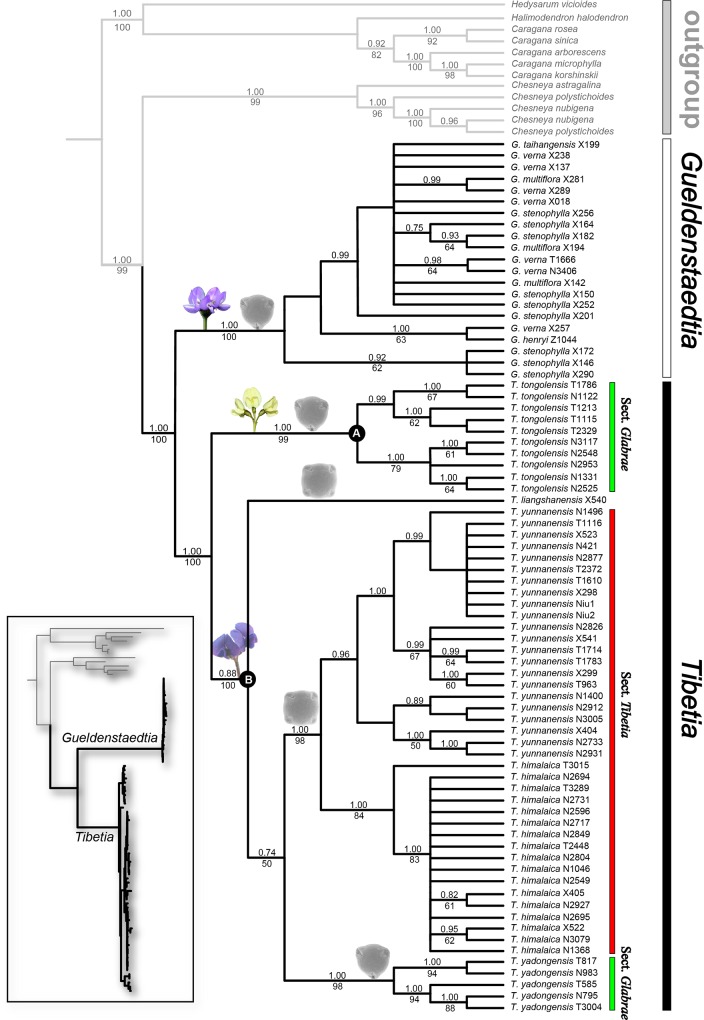
A Bayesian consensus tree of *Tibetia* and *Gueldenstaedtia* based on combined nuclear and chloroplast sequences (tree length = 1224 steps, CI = 0.81, and RI = 0.97). The bootstrap values in 1000 replicates are shown under the branches and Bayesian posterior probabilities higher than 95% are indicated above the lines. Maximum likelihood topology is displayed at the bottom left.

The aligned ITS matrix was composed of 720 base pairs (bp) with 127 phylogenetically informative sites. The combined plastid matrix was 4822 bp in length with 475 informative sites. Gaps were treated as missing data. Incongruence was detected between nuclear and plastid sequences (ILD, P < 0.05), thus whether the conflicting data sets could be combined in a simultaneous analysis is a complex and controversial decision [47,48]. In this case, both nuclear ITS and the chloroplast sequences data suggest the monophyly of *Tibetia* and *Gueldenstaedtia*, but the relationships among species within each of these clades are not clear (S1 and S2 Figs). However, the combined nuclear and chloroplast sequences data showed significant improvement in phylogenetic resolution, including strong support for species relationships within *Tibetia* (Fig 2), so we, therefore, combined all of the data for further phylogenetic analyses.

The combined nuclear and plastid sequence dataset was 5542 bp in length, with 602 positions that were parsimony-informative. Using the variable positions, more than one million MPTs were generated with a length of 1224 steps, a consistency index (CI) of 0.81 (CI excluding uninformative characters = 0.75), a retention index (RI) of 0.97, and a rescaled consistency index (RC) of 0.78. The ML, MP, and Bayesian analyses produced similar topologies. The Bayesian consensus tree based on combined nuclear and chloroplast sequences with PP and BP values are shown in Fig 2.

The monophyly of *Tibetia* and *Gueldenstaedtia* is strongly supported (PP = 1.00, BP = 100), and they are sister to each other (Fig 2). *Chesneya* is the closest relative of these two genera with high support (PP = 1.00, BP > 80). Phylogenetic relationships within *Gueldenstaedtia* remain unclear and different accessions from a single species are not grouped together. However, two major clades are robustly distinguished in *Tibetia* and all species are supported to be monophyletic with individual accessions clustered together (Fig 2). One clade includes *T*. *tongolensis* (clade A) as sister to all the rest species (clade B). Three subclades and one lineage are recognized in clade B corresponding to four distinct species of *T*. *yunnanensis*, *T*. *himalaica*, *T*. *yadongensis*, and *T*. *liangshanensis* (Fig 2).

The pairwise distances between *Tibetia* and *Gueldenstaedtia*, and among species of these two genera were estimated in the software PAUP* 4.10b10 (Table 2). The highest divergence (8.78% - 12.40%) was between *Tibetia* and *Gueldenstaedtia* in *psbA-trnH* intron sequences. The species within *Tibetia* showed a much higher level of intraspecific sequence variation compared with species in *Gueldenstaedtia*, ranging from twice (*psbA-trnH*) to ten times (combined chloroplast sequences; Table 2).

**Table 2:** Sequence characteristics of *Tibetia* and *Gueldenstaedtia*, and sequence divergence values, which were estimated with pairwise distance.

Characteristics	ITS	*matK*	*rbcL*	*psbA-trnH*	*trnL-F*	chloroplast	Combined
No. species sequenced	69	71	70	61	66	76	76
Missing data (%)	9.21	6.58	7.89	19.74	13.16	0	0
Sequence length (bp)	720	1726	1391	577	1128	4822	5542
Intra *Gueldenstaedtia* (%)	0–0.3	0–0.29	0–0.15	0–1.22	0–0. 12	0–0. 17	0–0. 16
Intra *Tibetia* (%)	0–1.57	0–0. 98	0–0. 72	0–2.40	0–1.12	0–1.71	0–1.21
Intergenera (%)	4.76–5.97	3.44–4.52	1.67–2.73	8.78–12.40	6.57–8.58	3.11–9.32	3.15–8.14

**Fig 3:**
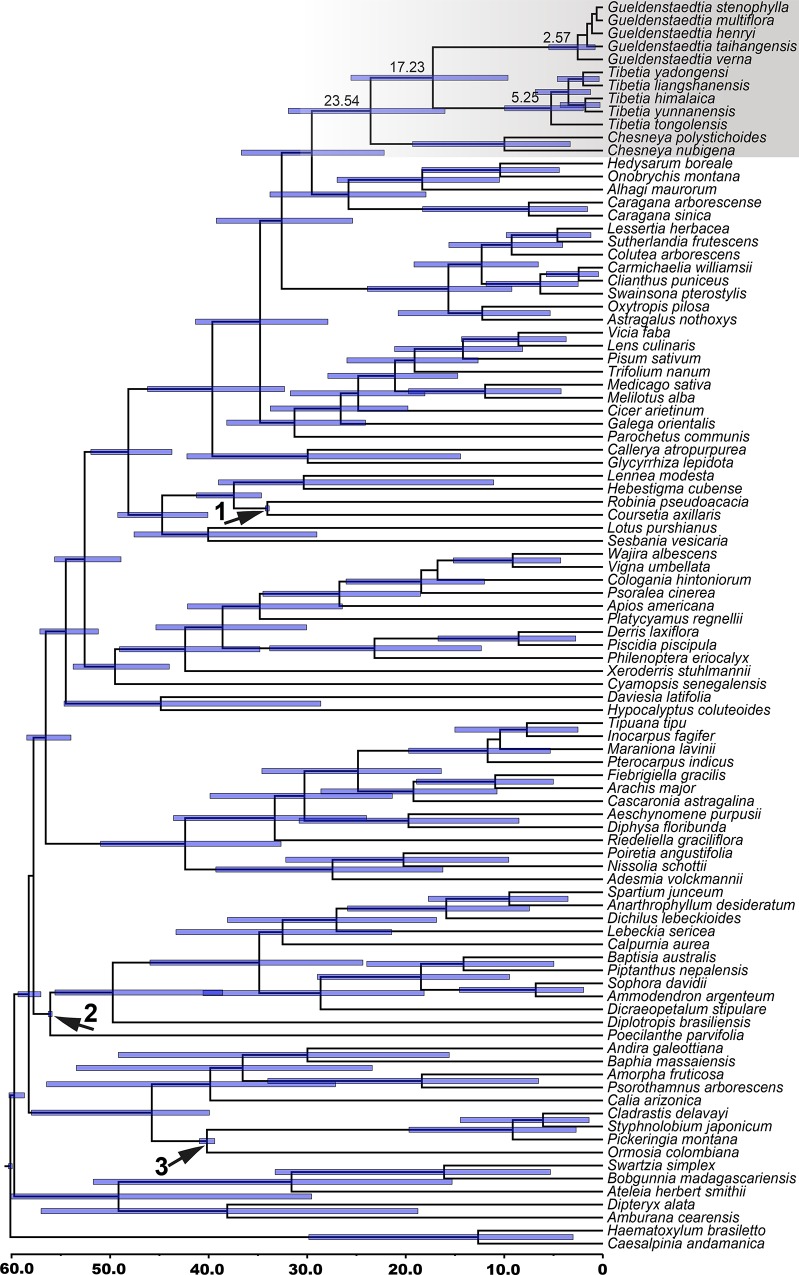
Chronogram of *Tibetia*, *Gueldenstaedtia* and other related taxa from the Papilionoideae based on *matK* data. Divergence times are shown using the computer program BEAST. The calibration nodes 1 (34±0.1 Ma), 2 (56±0.1 Ma), and 3 (40±0.4 Ma) are marked by arrows based on the fossil records. The root of the tree was set to no more than 60 Ma.

A total of 96 taxa covering almost the entire diversity of the Papilionoideae were selected for the dating analysis, based mainly on the framework at the subfamily level presented by Lavin et al. (2005). Bayesian dating with fossil calibrations suggested that *Gueldenstaedtia* and *Tibetia* diversified from each other at approximately 17.23 Ma (95% HPD: 9.61–25.52 Ma) in the early Miocene. The diversification of *Tibetia* species began around 5.25 Ma (95% HPD: 1.99–9.96 Ma) while the diversification of its sister genus *Gueldenstaedtia* was more recent and diverged at *ca*. 2.57 Ma (95% HPD: 0.79–5.49 Ma) (Fig 3).

## Discussion

### Phylogenetic status of *Tibetia* and *Gueldenstaedtia*

The monophyly of *Tibetia* is robustly supported by molecular phylogenetic analyses based on the combined nuclear ITS and cpDNA dataset with a comprehensive sampling (PP = 1.00, BP = 100, Fig 2), in agreement with previous morphological and taxonomical studies [3,22]. *Tibetia* are endemic to the Hengduan Mountain region (i.e. Sino-Himalayan subkingdom), and its prostrate habit and dense glandular hair might be an adaptation to the high-altitude plateau.

*Gueldenstaedtia* is distinguished from *Tibetia* and other relatives with a chromosomal number of 2n = 14, and is also confirmed to be monophyletic by the molecular evidence (PP = 1.00, BP = 100; Fig 2). The genus is mostly restricted in the Sino-Japanese subkingdom (Fig 1) and distributed widely in eurytopic habitats, and has morphological characters with continuous variations that have caused challenges in the identification of infrageneric relationships due to the instability in these traits. The difficulty of distinguishing species morphologically for the genus is probably reflected in the difficulty of resolving infrageneric relationships based on molecular phylogenetic analyses.

The sister relationship of these two genera is also suggested in this study with high support (PP = 1.00, BP = 100, Fig 2). Morphologically, they share many synapomorphic features, such as small herbaceous plants, stems that are almost absent, or with very short or many branches, pinnate leaves, and racemes composed of several yellow or purple papilionaceous flowers, calyx base symmetric or suboblique, wings usually palmately nerved, legume valves not twisting, keel petals half as long as wings and style shorter than or as long as ovary [6,22,49]. The two genera are distributed throughout the Sino-Himalayan and Sino-Japanese subkingdoms in eastern Asia and flower from May to June. Based on this information, some scholars classified *Tibetia* and *Gueldenstaedtia* as one genus [50–53]. It is controversial to separate *Tibetia* from *Gueldenstaedtia* [4,50,52]; however, our phylogenetic results strongly support the monophyly of these genera. The two genera are also morphologically and cytologically distinct (Table 1). For example, *Gueldenstaedtia* always has lateral and separated stipules, non-spotted seeds, and chromosome base number of x = 7, while *Tibetia* has amplexicaul and united stiples, irregular spotted seeds, and chromosome base number of x = 8. Together with our phylogenetic results and the morphological and cytological characteristics, we support the separation of *Tibetia* as independent genus and as a sister group to *Gueldenstaedtia*.

*Chesneya* has been suggested to be one of the closest relatives of *Tibetia* and *Gueldenstaedtia* because of the cytological (chromosome number of *Chesneya* is x = 8) and molecular evidence [11,12,54]. Our data well recognize a clade including *Gueldenstaedtia*, *Tibetia*, and *Chesneya* species (PP = 1.00, BP = 99) and suggest *Chesneya* is sister to *Tibetia* and *Gueldenstaedtia*. This agreed with previous molecular analyses concerning these genera [10,12,55]. Furthermore, *Chesneya* species are perennial herbs with thick roots, lignification, very short stems, leaves imparipinnate, and similar pods to *Gueldenstaedtia* and *Tibetia* species, supporting their close relationship. Ranjbar and Karamian (2003) established the new tribe, Caraganeae, which joins *Caragana*, *Halimodendron*, *Calophaca*, *Chesneya*, and *Gueldenstaedtia*. This new tribe is morphologically characterized by the pedicel asymmetrically attached to a slightly gibbous ± tubular calyx (except *Halimodendron*), the valves of the pod generally twist upon dehiscence (except *Halimodendron*), and opening calyx during fruiting [13]. However, Duan et al. [10] did not support the monophyletic status of the tribe based on ITS, *matK*, *trnL-F*, and *psbA-trnH* sequences. This tribe is separated into two monophyletic subtribes, the Caraganean clade (including *Caragana*, *Halimodendron*, and *Calophaca*) and the Chesneyean clade (consisting of *Chesneya*, *Gueldenstaedtia*, and *Tibetia*).

### Phylogenetic relationships within each genus

Two major lineages corresponding to flower color were well recognized in *Tibetia* (Fig 2). The first includes only *T*. *tongolensis* with yellow flowers and a glabrous ovary (clade A), which is distinct from all others in the genus, which have dark purple, violet, or blue flowers and a pubescent ovary (clade B). *T*. *tongolensis* is very unique with its yellow flowers in *Tibetia*. Additionally, the other species in *Tibetia* and their sister genus, *Gueldenstaedtia*, are all purple-flowered; therefore, the yellow flower color is apparently a derived character state in this group.

Three lineages were well distinguished in the clade B, and relationships among the three lineages are unclear (Fig 2). The first includes *T*. *yunnanensis* and *T*. *himalaica* from sect. *Tibetia* which is morphologically characterized by 4-colporate pollen grains and stipules acute at the apex. However, some individual samples from *T*. *yunnanensis* and *T*. *himalaica* appear within different clades on the plastid or nuclear tree (S1 and S2 Figs), which may be caused by DNA mutation or random introgression events. Another clade includes only *T*. *yadongensis* with 3-cloporate pollen grains, belonging to sect. *Glabrae* [22,49]. The third lineage includes only *T*. *liangshanensis*, which was originally treated as *T*. *forrestii* (Ali) P. C. Li in Zhu [22] However, phylogenetic and morphological evidences supported the recognition of *T*. *liangshanensis* as a different species from *T*. *yunnanensis* [8], which has significantly different morphological characters, including more leaflets per pinnate leaf, a smooth abaxial epidermis, and free stipules, *vs*. one to four leaflets per pinnate leaf, a squamose abaxial epidermis, and stipules connecting at the base in *T*. *yunnanensis* [8].

Although the monophyly of *Gueldenstaedtia* is robustly supported, the infrageneric relationships are uncertain as shown with extremely short branch lengths and low resolutions (Fig 2). This might be a result of the recent origin of *Gueldenstaedtia*, without enough time for sequence variation to accumulate. The circumscription of species within *Gueldenstaedtia* is usually based on whether the stem is lignified or not, the leaflet is broad or narrow, the flowers are many or few, and the plants pubescent or glabrous [4]. However, these characteristics are not dependable to discriminate different species that may have continuous morphological variation. It is possible to consider *Gueldenstaedtia* as a species complex with abundant continuous characters.

### Biogeographic differentiation pattern within eastern Asia

The divergence of *Tibetia* from *Gueldenstaedtia* was estimated at about 17.23 Ma (95% HPD: 9.61–25.52 Ma) in the early Miocene. Many other plant groups from the QTP and adjacent regions have also been estimated to have originated or diverged from their relatives around this time. For example, the diversification of the cushion-like “*Androsace* group” in the QTP from other sister genera was speculated at 18.02 Ma [56]. The explosive radiation of the *Ligularia–Cremanthodium—Parasenecio* complex also occurred mostly within the last 20 Ma likewise, perhaps effected by the major uplifts of the QTP in the early Miocene [57]. Most species of the temperate Asian genus *Caragana* are distributed on the QTP and in northwestern China. *Caragana* species diversified at about 16–14 Ma, and were also presumably triggered by the QTP uplift at 21–17 Ma [58]. The Sino-Himalayan endemic genus *Cyananthus*, with an estimated origin 23–12 Ma, may be another case of diversification in response to the Himalayan uplifts [59]. The QTP went through roughly four intense uplifts in total, i.e., 25–17 Ma, 15–13 Ma, 8–7 Ma, and 3.5–1.6 Ma [60–63]. The Himalayan uplift around 25–17 Ma was one of the most significant events [64], which is considered to have triggered the diversification of many plant and animal taxa in this area [57,58,65,66]. Similar to the taxa discussed above that originated and diversified during the QTP uplift, the split of *Tibetia* and *Gueldenstaedtia* that occurred about 17 Ma might be also have been driven by this extensive architectonical movement during the early Miocene.

The flora of eastern Asia is subdivided into the Sino-Himalayan and Sino-Japanese subkingdoms [16,17]. The biogeographic pattern of *Tibetia* and *Gueldenstaedtia* species mostly corresponds to these two subkingdoms. The Sino-Himalayan *Tibetia* shows a much higher level of infrageneric divergence than the Sino-Japanese *Gueldenstaedtia* (Table 2). *Tibetia* diversified in the Sino-Himalayan region at *ca*. 5.25 Ma in late Miocene, which has subsequently undergone a significant change in topography and climate corresponding to the phased uplifts of the Himalayas [67]. This region is characterized by rich biodiversity, with many evolutionary radiations, and diversification of many plant groups associated with the varied isolated habitats and mountains [68–70]. In contrast, *Gueldenstaedtia* exhibits very low morphological variation and genetic differentiation. The genus is distributed in northern and central China all the way to Siberia, a region dominated by vast plains, which has rarely been affected by geological events. The physical environment has remained relatively stable and homogenous with fewer geographical barriers and weaker divergent selection pressures, which could explain the low molecular differentiation for *Gueldenstaedtia*.

## Supporting Information

S1 Fig Bayesian consensus tree of *Tibetia* and *Gueldenstaedtia* based on nuclear ITS sequences. (TIF)

S2 Fig Bayesian consensus tree of *Tibetia* and *Gueldens**t**aedtia* based on combined plastid sequences. (TIF)

S1 Table Species, voucher with collection locality, geographical coordinates, and GenBank accession number for taxa included in this study. (DOCX)
