# Supplementary figures and images for: Molecular Phylogeny of Gueldenstaedtia and Tibetia (Fabaceae) and Their Biogeographic Differentiation within Eastern Asia
Source: PLoS One. 2016 Sep 15;11(9):e0162982. doi: 10.1371/journal.pone.0162982 (PMC5025100; doi:10.1371/journal.pone.0162982)

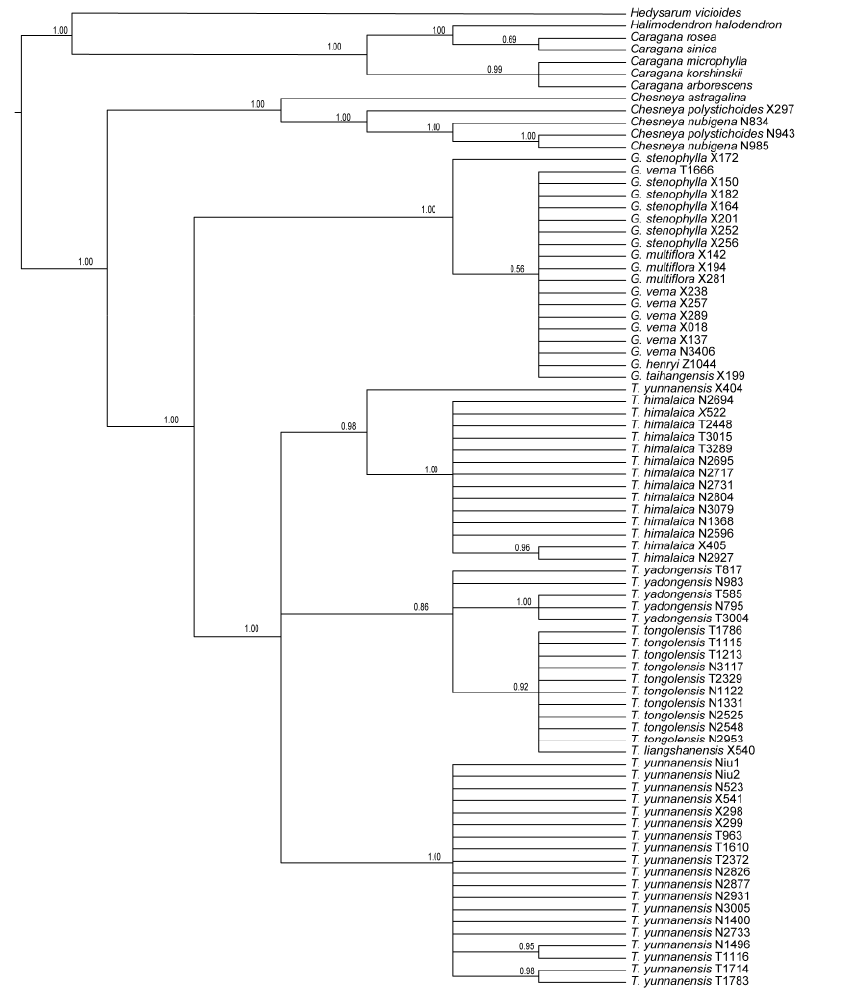

Supplement: S1 Fig — (TIF) [file pone.0162982.s001.tif]

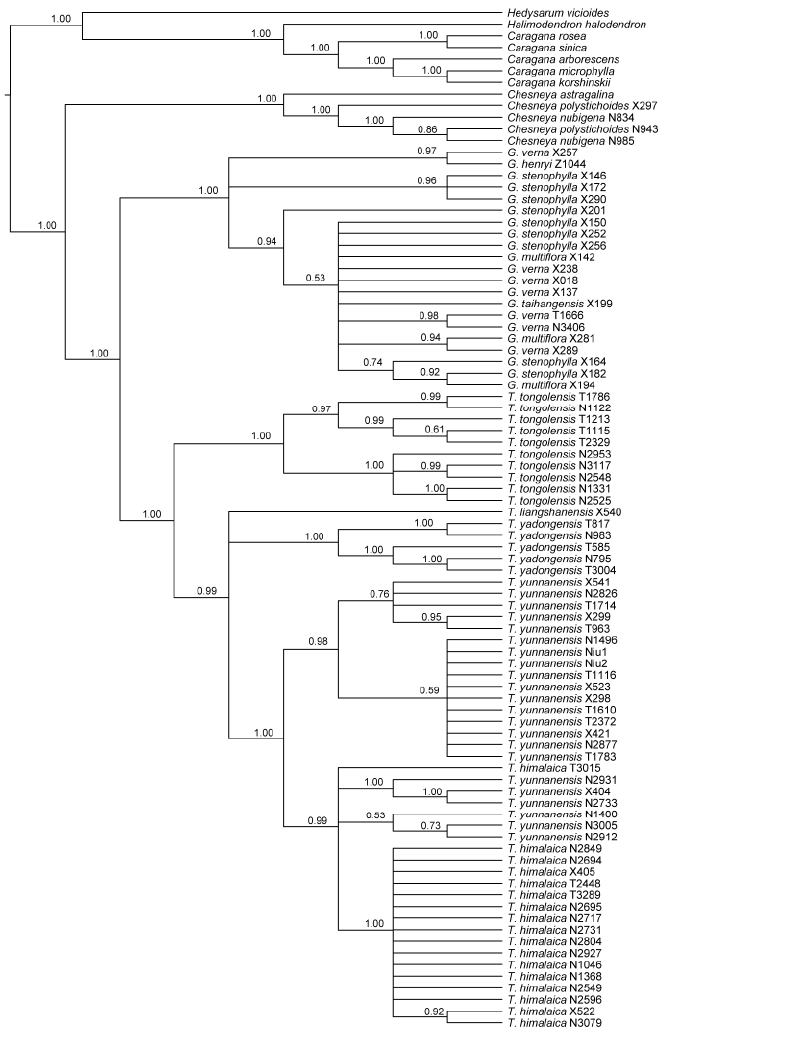

Supplement: S2 Fig — (TIF) [file pone.0162982.s002.tif]
