# Supplementary material for: Molecular Phylogeny of Gueldenstaedtia and Tibetia (Fabaceae) and Their Biogeographic Differentiation within Eastern Asia
Source: PLoS One. 2016 Sep 15;11(9):e0162982. doi: 10.1371/journal.pone.0162982 (PMC5025100; doi:10.1371/journal.pone.0162982)
Supplement: S1 Table — (DOCX) [file pone.0162982.s003.docx]

| Taxa | No. | Voucher | Locality | Geographical coordinates | ITS | *matK* | *rbcL* | *psbA-trnH* | *trnL-F* |
| --- | --- | --- | --- | --- | --- | --- | --- | --- | --- |
| *G. verna* | N3406 | Nie 3406 | Sichuan | 100°10′18.74″E; 26°15′7.33″N | KX021412 | KX021487 | KX021562 | KX021702 | KX021636 |
| *G. verna* | T1666 | Tibet 1666 | Yunnan, Lijiang | 99°57'35.4''E; 26°56′0.06″N | KX021447 | KX021524 | KX021596 | ------ | KX021666 |
| *G. verna* | X018 | Xie 018 | Shaanxi, Baoji | 107°45′9.45″E; 34°09′37.89″N | KX021403 | KX021478 | KX021553 | KX021696 | KX021627 |
| *G. verna* | X137 | Xie 137 | Shaanxi, Baoji | 107°45′5.42″E; 34°05′15.89″N | KX021404 | KX021479 | KX021554 | KX021697 | KX021628 |
| *G. verna* | X238 | Xie 238 | Shandong, Yanzhou | 116°49′20.4″E; 36°31′36.11″N | KX021392 | KX021466 | KX021541 | KX021685 | KX021615 |
| *G. verna* | X257 | Xie 257 | Henan, Luoyang | 111°51′48.1″E; 34°06′19.2″N | KX021395 | KX021469 | KX021544 | KX021687 | KX021618 |
| *G. verna* | X289 | Xie 289 | Henan, Luoyang | 111°36′10.5″E; 33°46′58.1″N | KX021397 | KX021471 | KX021546 | KX021689 | KX021620 |
| *G. stenophylla* | X146 | Xie 146 | Gansu, Pingliang | 106°57′31.25″E; 34°23′37.62″N | ------ | KX021458 | KX021533 | KX021677 | KX021607 |
| *G. stenophylla* | X150 | Xie 150 | Gansu, Heshui | 108°40′54.23″E; 36°07′5.57″N | KX021386 | KX021460 | KX021535 | KX021679 | KX021609 |
| *G. stenophylla* | X164 | Xie 164 | Shanxi, Changzhi | 112°33′46.4″E; 36°47′20.7″N | KX021390 | KX021464 | KX021539 | KX021683 | KX021613 |
| *G. stenophylla* | X172 | Xie 172 | Hebei, Handan | 113°40′43.4″E; 36°33′17.0″N | KX021387 | KX021461 | KX021536 | KX021680 | KX021610 |
| *G. stenophylla* | X182 | Xie 182 | Hebei, Handan | 113°45′5.00″E; 36°48′17.33″N | KX021388 | KX021462 | KX021537 | KX021681 | KX021611 |
| *G. stenophylla* | X201 | Xie 201 | Shandong, Taian | 117°09′07.4″E; 36°13′28.4″N | KX021391 | KX021465 | KX021540 | KX021684 | KX021614 |
| *G. stenophylla* | X252 | Xie 252 | Shandong, Zoucheng | 117°07′19.9″E; 35°24′04.2″N | KX021393 | KX021467 | KX021542 | ------ | KX021616 |
| *G. stenophylla* | X256 | Xie 256 | Shandong, Zoucheng | 116°48′55.63″E; 35°13′20.98″N | KX021394 | KX021468 | KX021543 | KX021686 | KX021617 |
| *G. stenophylla* | X290 | Xie 290 | Henan, Jiaozuo | 113°12′18.4″E; 35°03′35.6″N | ------ | KX021472 | KX021547 | KX021690 | KX021621 |
| *G. multiflora* | X142 | Xie 142 | Shaanxi, Baoji | 106°57′31.25″E;34°23′37.62″N | KX021385 | KX021459 | KX021534 | KX021678 | KX021608 |
| *G. multiflora* | X194 | Xie 194 | Hebei, Wuan | 113°48′31.8″E; 36°55′27.3″N | KX021389 | KX021463 | KX021538 | KX021682 | KX021612 |
| *G. multiflora* | X281 | Xie 281 | Henan, Luoyang | 111°36′10.5″E; 33°46′58.1″N | KX021396 | KX021470 | KX021545 | KX021688 | KX021619 |
| *G. henryi* | Z1044 | Zhou1044 | Hubei, Shiyan | 110°25′35.00″E; 32°59′25.55″N | KX021446 | KX021523 | KX021595 | KX021741 | KX021665 |
| *G. taihangensis* | X199 | Xie 199 | Hebei, Wuan | 113°48′31.8″E; 36°55′27.3″N | KX021405 | KX021480 | KX021555 | KX021698 | KX021629 |
| *T. tongolensis* | N1122 | Nie 1122 | Sichuan, Muli | 101°09′56″E; 28°08′25″N | KX021438 | KX021515 | KX021586 | KX021732 | KX021660 |
| *T. tongolensis* | N1331 | Nie 1331 | Sichuan, Kangding | 101°58′48″E; 29°55′48″N | KX021439 | KX021516 | KX021587 | KX021733 | ------ |
| *T. tongolensis* | N2525 | Nie 2525 | Sichuan, Kangding | 101°34′07.6″E; 30°32′40.3″N | KX021443 | KX021520 | KX021591 | KX021737 | KX021663 |
| *T. tongolensis* | N2548 | Nie 2548 | Sichuan, Kangding | 101°34′07.6″E; 30°32′40.3″N | KX021444 | KX021521 | KX021592 | KX021738 | ------ |
| *T. tongolensis* | N2953 | Nie 2953 | Sichuan, Kangding | 101°28′44.2″E; 29°24′02.2″N | KX021432 | KX021508 | KX021579 | KX021725 | KX021655 |
| *T. tongolensis* | N3117 | Nie 3117 | Sichuan, Kangding | 101°44′43.6″E; 30°11′09.1″N | KX021401 | KX021476 | KX021551 | KX021694 | KX021625 |
| *T. tongolensis* | T1115 | Tibet 1115 | Yunnan, Shangri-La | 99°49'12.7''E; 27°27' 58.6″N | KX021449 | KX021526 | KX021598 | ------ | KX021668 |
| *T. tongolensis* | T1213 | Tibet 1213 | Yunnan, Shangri-La | 99°54' 23.7''E; 28°08'32.2″N | KX021454 | KX021529 | KX021603 | ------ | KX021673 |
| *T. tongolensis* | T1786 | Tibet 1786 | Sichuan, Muli | 101°09'49.6''E; 28°08'28.6″N | KX021448 | KX021525 | KX021597 | ------ | KX021667 |
| *T. tongolensis* | T2329 | Tibet 2329 | Yunnan, Shangri-La | 99°12'28.8''; 23°39'03.9″N | KX021402 | KX021477 | KX021552 | KX021695 | KX021626 |
| *T. yunnanensis* | Niu1 | Niu 080629 | Yunnan, Shangri-La | ------ | KX021451 | KX021527 | KX021600 | ------ | KX021670 |
| *T. yunnanensis* | Niu2 | Niu 080702 | Yunnan, Shangri-La | ------ | KX021452 | KX021528 | KX021601 | ------ | KX021671 |
| *T. yunnanensis* | X298 | Xie 298 | Yunnan, Lijiang | 100°11′53.09″E; 27°0′0.40″N | KX021414 | KX021489 | ------ | KX021704 | ------ |
| *T. yunnanensis* | N1400 | Nie 1400 | Sichuan, Daocheng | 100°16′03″E; 28°49′44″N | KX021441 | KX021518 | KX021589 | KX021735 | ------ |
| *T. yunnanensis* | N1496 | Nie 1496 | Yunnan, Shangri-La | 99°51′42″E; 27°35′55″N | KX021442 | KX021519 | KX021590 | KX021736 | KX021662 |
| *T. yunnanensis* | N2733 | Nie 2733 | Sichuan, Litang | 100°32′10.1″E; 29°42′33.7″N | KX021426 | KX021500 | KX021573 | KX021717 | KX021648 |
| *T. yunnanensis* | N2826 | Nie 2826 | Sichuan, Daocheng | 100°30′40.3″E; 29°03′20.8″N | KX021428 | KX021502 | ------ | KX021719 | KX021650 |
| *T. yunnanensis* | N2877 | Nie 2877 | Sichuan, Kangding | 100°15′29.9″E; 28°43′32.7″N | KX021429 | KX021504 | KX021576 | KX021721 | KX021652 |
| *T. yunnanensis* | N2912 | Nie 2912 | Sichuan, Daocheng | 100°18′43.5″E; 28°56′38.7″N | ------ | KX021505 | KX021577 | KX021722 | ------ |
| *T. yunnanensis* | N2931 | Nie 2931 | Sichuan, Kangding | 101°31′04.9″E; 29°46′54.0″N | KX021431 | KX021507 | ------ | KX021724 | KX021654 |
| *T. yunnanensis* | N3005 | Nie 3005 | Sichuan, Jiulong | 101°29′56.1″E; 29°20′03.6″N | KX021433 | KX021509 | KX021580 | KX021726 | ------ |
| *T. yunnanensis* | T963 | Tibet 963 | Yunnan, Lijiang | ------ | KX021416 | KX021491 | KX021564 | KX021706 | KX021638 |
| *T. yunnanensis* | T1116 | Tibet 1116 | Yunnan, Shangri-La | 99°49'12.7''E; 27°27' 58.6″N | KX021456 | KX021531 | KX021605 | ------ | KX021675 |
| *T. yunnanensis* | T1610 | Tibet 1610 | Yunnan, Weixi | 99°25'35.4''E; 27°10'237″N | KX021417 | KX021492 | KX021565 | KX021707 | KX021639 |
| *T. yunnanensis* | T1714 | Tibet 1714 | Sichuan, Muli | 101°13'24.7''E; 27°41'144″N | KX021455 | KX021530 | KX021604 | ------ | KX021674 |
| *T. yunnanensis* | T1783 | Tibet 1783 | Sichuan, Muli | 101°09'49.6''E; 28°08'28.6″N | KX021453 | ------ | KX021602 | ------ | KX021672 |
| *T. yunnanensis* | T2372 | Tibet 2372 | Yunnan, Shangri-La | 99°42' 45.6''E; 27°36' 52.6″N | KX021418 | ------ | ------ | KX021708 | KX021640 |
| *T. yunnanensis* | X299 | Xie 299 | Yunnan, Lijiang | 100°12'23.09″E; 27°01' 09.40″N | KX021415 | KX021490 | ------ | KX021705 | ------ |
| *T. yunnanensis* | X404 | Xie 404 | Sichuan, Hongyuan | 102°36′41.1″E; 32°00′40.3″N | KX021406 | KX021481 | KX021556 | KX021699 | KX021630 |
| *T. yunnanensis* | N421 | Nie 421 | Yunnan, Lijiang | 99°23'55.74"E; 27°12'01.92"N | ------ | KX021496 | KX021569 | KX021713 | KX021644 |
| *T. yunnanensis* | X523 | Xie 523 | Sichuan, Jiulong | 101°32′4.96″; 29°17′24.25″ | KX021409 | KX021484 | KX021559 | ------ | KX021633 |
| *T. yunnanensis* | X541 | Xie 541 | Sichuan, Zhaojue | 102°34′0.83″; 27°52′48.29″ | KX021411 | KX021486 | KX021561 | ------ | KX021635 |
| *T. himalaica* | N1046 | Nie 1046 | Xizang, Linzhi | 93°01′53.0″E; 28°49′27.6″N | ------ | KX021514 | KX021585 | KX021731 | KX021659 |
| *T. himalaica* | N1368 | Nie 1368 | Sichuan, Litang | 100°52′48″E; 29°59′50″N | KX021440 | KX021517 | KX021588 | KX021734 | KX021661 |
| *T. himalaica* | N2549 | Nie 2549 | Sichuan, Kangding | 101°34′07.6″E; 30°32′40.3″N | ------ | KX021522 | KX021593 | KX021739 | ------ |
| *T. himalaica* | N2596 | Nie 2596 | Sichuan, Yajiang | 100°51′33.9″E; 30°00′42.6″N | KX021445 | ------ | KX021594 | KX021740 | KX021664 |
| *T. himalaica* | N2694 | Nie 2694 | Sichuan, Litang | 99°51′16.2″E; 29°50′48.8″N | KX021400 | KX021475 | KX021550 | KX021693 | KX021624 |
| *T. himalaica* | N2695 | Nie 2695 | Sichuan, Litang | 99°51′16.2″E; 29°50′48.8″N | KX021423 | KX021497 | KX021570 | KX021714 | KX021645 |
| *T. himalaica* | N2717 | Nie 2717 | Sichuan, Litang | 100°32′10.1″E; 29°42′33.7″N | KX021424 | KX021498 | KX021571 | KX021715 | KX021646 |
| *T. himalaica* | N2731 | Nie 2731 | Sichuan, Litang | 100°32′10.1″E; 29°42′33.7″N | KX021425 | KX021499 | KX021572 | KX021716 | KX021647 |
| *T. himalaica* | N2804 | Nie 2804 | Sichuan, Xiangcheng | 99°44′30.9″E; 29°00′05.2″N | KX021427 | KX021501 | KX021574 | KX021718 | KX021649 |
| *T. himalaica* | N2849 | Nie 2849 | Sichuan, Daocheng | 100°30′40.3″E; 29°03′20.8″N | ------ | KX021503 | KX021575 | KX021720 | KX021651 |
| *T. himalaica* | N2927 | Nie 2927 | Sichuan, Kangding | 101°31′04.9″E; 29°46′54.0″N | KX021430 | KX021506 | KX021578 | KX021723 | KX021653 |
| *T. himalaica* | N3079 | Nie 3079 | Sichuan, Kangding | 101°36′48.9″E; 29°27′43.8″N | KX021434 | KX021510 | KX021581 | KX021727 | KX021656 |
| *T. himalaica* | T2448 | Tibet 2448 | Yunnan, Shangri-La | 99°38' 37''E; 27°37' 47″N | KX021419 | ------ | ------ | KX021709 | KX021641 |
| *T. himalaica* | T3015 | Tibet 3015 | Xizang, Linzhi | 94°57'28.08"E; 30°00'31.44"N | KX021421 | KX021494 | KX021567 | KX021711 | KX021642 |
| *T. himalaica* | T3289 | Tibet 3289 | Xizang, Lasa | 94°13'4.38"E; 30°00'14.10N | KX021422 | KX021495 | KX021568 | KX021712 | KX021643 |
| *T. himalaica* | X405 | Xie 405 | Sichuan, Hongyuan | 102°37′02.8″E; 32°01′17.0″N | KX021407 | KX021482 | KX021557 | KX021700 | KX021631 |
| *T. himalaica* | X522 | Xie 522 | Sichuan, Jiulong | 101°32′4.96″; 29°17′24.25″ | KX021408 | KX021483 | KX021558 | KX021701 | KX021632 |
| *T. yadongensis* | N795 | Nie 795 | Xizang, Cuona | 91°46′32.8″E; 27°50′53.8″N | KX021398 | KX021473 | KX021548 | KX021691 | KX021622 |
| *T. yadongensis* | N983 | Nie 983 | Xizang, Nielamu | 85°57′11.7″E; 28°02′09.9″N | KX021436 | KX021512 | KX021583 | KX021729 | ------ |
| *T. yadongensis* | T585 | Tibet 585 | Xizang, Linzhi | ------ | KX021457 | KX021532 | KX021606 | ------ | KX021676 |
| *T. yadongensis* | T817 | Tibet 817 | Xizang, Jilong | ------ | KX021450 | ------ | KX021599 | ------ | KX021669 |
| *T. yadongensis* | T3004 | Tibet 3004 | Xizang, Linzhi | 94°57'28.08"E; 30°00'31.44"N | KX021420 | KX021493 | KX021566 | KX021710 | ------ |
| *T. liangshanensis* | X540 | Xie 540 | Sichuan, Zhaojue | 102°34′0.83″; 27°52′48.29″ | KX021410 | KX021485 | KX021560 | ------ | KX021634 |
| *Chesneya nubigena* | N834 | Nie 834 | Xizang, Cuona | 91°52′48.6″E; 27°58′24.0″N | KX021399 | KX021474 | KX021549 | KX021692 | KX021623 |
| *Ch.nubigena* | N985 | Nie 985 | Xizang, Nielamu | 85°57′11.7″E; 28°02′09.9″N | KX021437 | KX021513 | KX021584 | KX021730 | KX021658 |
| *Ch.polystichoides* | N943 | Nie 943 | Xizang, Yadong | 88°53′23.4″E; 27°22′52.5″N | KX021435 | KX021511 | KX021582 | KX021728 | KX021657 |
| *Ch. polystichoides* | X297 | Xie 297 | Sichuan, Daxueshan | ------ | KX021413 | KX021488 | KX021563 | KX021703 | KX021637 |
| *Ch.astragalina* |  | Genebank | ------ |  | AB051906 | ------ | ------ | JF409800 | AB287413 |
| *Halimodendron halodendron* |  | Genebank | ------ |  | FJ537289 | ------ | Z95536 | ------ | GQ890304 |
| *Caragana arborescens* |  | Genebank | ------ |  | L10798 | AF142737 | FJ537211 | GU396766 | GQ890296 |
| *C. Korshinskii* |  | Genebank | ------ |  | FJ537266 | AY189778 | FJ537215 | ------ | ------ |
| *C. microphylla* |  | Genebank | ------ |  | FJ537264 | AY189780 | FJ537214 | ------ | AY633701 |
| *C. sinica* |  | Genebank | ------ |  | FJ537284 | HM049541 | FJ537233 | GU396767 | DQ914754 |
| *C. rosea* |  | Genebank | ------ |  | FJ537272 | HM049543 | FJ537221 | GU396769 | DQ914743 |
| *Hedysarum vicioides* |  | Genebank | ------ |  | HM142304 | HM142267 | HM142227 | ------ | ------ |
